# Clusterin silencing inhibits proliferation and reduces invasion in human laryngeal squamous carcinoma cells

**DOI:** 10.1186/1477-7819-12-124

**Published:** 2014-04-26

**Authors:** Qianjin Wang, Weiyan Cao, Quancai Su, Zimin Liu, Lin Zhang

**Affiliations:** 1 Department of Otolaryngology, Ju-nan County People’s Hospital, Ju-nan, Linyi 276600, China; 2 Department of Obstetrics, Qingdao Central Hospital, Qingdao 266012, China; 3 Department of Anesthesiology, the Affiliated Hospital of Qingdao University, Qingdao 266003, China; 4 Department of Ontology, the Affiliated Hospital of Qingdao University, Qingdao 266003, China

**Keywords:** Laryngeal squamous carcinoma, Clusterin, Proliferation, Apoptosis, Invasion, Gene treatment

## Abstract

**Background:**

Clusterin is, in its major form, a secreted heterodimeric disulfide-linked glycoprotein (sCLU), which plays important roles in cell survival and death. In laryngeal squamous cell carcinomas (LSCC), sCLU is up-regulated and its expression is related to the invasiveness of these tumors. The purpose of this study was to explore the inhibiting role of sCLU gene silence in the invasive ability and growth of Hep-2 human laryngeal squamous carcinoma cells (Hep-2) by transfection of short hairpin RNA expression plasmids against sCLU (sCLU-shRNA) (*in vivo*) or small interference RNA (sCLU-siRNA) (*in vitro*).

**Methods:**

sCLU-siRNA and the control siRNA were transfected into Hep-2 cells using Lipofectamine 2000. RT-PCR and Western blot were used to detect the effect of siRNA transfection on sCLU mRNA and sCLU protein expression. The invasive activity of sCLU-siRNA-transfected Hep-2 cells was measured with the modified Boyden chamber assay and wound healing assay. The effects of sCLU-siRNA on cell proliferation were evaluated by MTT assay. Apoptosis was measured by Annexin V-fluorescein isothiocyanate (FITC)/propidium iodide (PI) double-staining methods. We next evaluated the effects of sCLU silencing by sCLU-shRNA transfection *in vivo* on tumor growth and metastatic properties to the lung. Terminal deoxytransferase-mediated dUTP nick end labeling (TUNEL) staining was used to observe the apoptosis in the xenografts.

**Results:**

It showed that siRNA-mediated down-regulation of sCLU expression in Hep-2 cells significantly inhibited cell proliferation and promoted apoptosis *in vitro*. Furthermore, siRNA-mediated down-regulation of sCLU expression decreases *in vitro* cell migration and invasion ability. *In vivo*, the average volume of tumors in the sCLU-shRNA transfected group was significantly lower than in the control group (*P* <0.01), and the significant apoptosis detected with TUNEL was indicated in the sCLU-shRNA transfected groups (*P* <0.05). Significantly, we found that sCLU-shRNA could exert marked inhibition of the lung metastasis of Hep-2 cells in nude mice *in vivo*.

**Conclusions:**

sCLU gene silence can inhibit invasion and growth of LSCC. sCLU may provide a potential therapeutic target against human LSCC.

## Background

Laryngeal cancer is the 11^th^ most common cancer worldwide. Laryngeal squamous cell carcinomas (LSCC) represent approximately 85 to 90% of all the malignant tumors of the larynx [1]. Although early-stage laryngeal cancer is often cured by surgery or radiotherapy, for the majority of patients with the advanced disease, the outcome has not improved in the last three decades. In addition, surgery might lead to complete or partial loss of vocal function and many patients have to maintain a tracheal cannula for life due to total laryngectomy. Therefore, a better understanding of the molecular mechanisms of LSCC progression and a new strategy for the treatment of LSCC are in urgent demand.

Clusterin is, in its major form, a secreted heterodimeric disulfide-linked glycoprotein (sCLU). It was first linked to cell death in the rat ventral prostate after androgen deprivation [2]. sCLU overexpression reaches a maximum at three to four days post-castration and coincides with the onset of massive cell death [3]. The sCLU level also rises in various malignant tumors, including gastric cancer [4], ovarian cancer [5], breast cancer [6], bladder cancer [7], colorectal cancer [8], hepatocellular carcinoma [9], prostate cancer [10] and laryngeal squamous cell carcinomas [11]. In these cancers, sCLU overexpression has been reported to be closely associated with cancer development and progression. Introducing the sCLU gene into renal cell carcinoma cells or hepatoma cells enhances their metastatic potential and causes enhanced formation of metastatic nodules in experimental animals [9,12]. Moreover, small interference RNA-mediated sCLU gene silencing inhibited invasion and metastasis in breast cancer cells [13,14], lung cancer cells [15,16] and prostate cancers [17].

sCLU has also been described as an anti-apoptotic factor. Miyake *et al*. [18] have demonstrated that the overexpression of sCLU in human androgen-responsive prostate cancer cells (LNCaP) by stable transfection rendered them highly resistant to androgen ablation, and the introduction of antisense testosterone-repressed message-2 oligodeoxynucleotide therapy in the Shionogi tumor model induces apoptosis and tumor regression. Moreover, small interference RNA-mediated sCLU gene silencing in osteosarcoma and breast cancer induces significant reduction of cellular growth and increases cellular apoptosis [19-21].

Previous study found sCLU was overexpressed in laryngeal carcinomas, and clusterin expression was significantly related to the degree of local invasion [11]. However, whether sCLU may be a target for the treatment of laryngeal carcinomas has not been elucidated. In the present study, we sought to investigate the effect of sCLU expression inhibition by using small interfering RNA (siRNA) and short hairpin RNA expression plasmids (shRNA) in Hep-2 cells on invasion, metastasis, apoptosis and proliferation *in vitro* and *in vivo*. Our findings indicate that knocking down the sCLU protein in Hep-2 cells by using siRNA induces growth retardation that is accompanied by higher rates of spontaneous endogenous apoptosis *in vitro*, and decreases *in vitro* cell migration and invasion ability. Moreover, sCLU silencing by using shRNA inhibits *in vivo* lung metastasis, tumor growth and induces apoptosis.

## Methods

### Cell culture

Laryngeal carcinoma Hep-2 cells (ATCC) were cultured in RPMI-1640 medium (GIBCO/BRL, Gaithersburg, Md, USA) supplemented with 10% new-born calf serum (GIBCO/BRL) with 100 U/ml penicillin and 100 μg/ml streptomycin at 37°C in a homeothermic incubator with a 5% CO_2_ atmosphere. The medium was changed every two or three days.

### siRNA transfection

Hep-2 cells suspended in DMEM with 10% FBS were added to each well of six-well plates. The plates were incubated at 37°C in a humidified atmosphere of 5% CO_2_. After the cell confluence reached 80% in each well, 50 nmol/L of sCLU-siRNA: AUGCCCUGUCUUACUGUCA or scramble sequences and 10 μL of LipofectAMINE 2000 (Invitrogen,Shanghai,China) were added to Opti-MEM (Life Technologies,Beijing,China) and mixed. After incubation, the siRNA and LipofectAMINE 2000 solutions were gently mixed and added to the plates. Each plate was incubated for 48 h until it was ready for further assay. The knockdown effect was verified by RT-PCR and Western blot analysis.

### Stable shRNA transfection

Short hairpin RNA against sCLU RNA (sCLU-shRNA) and control plaismid were purchased from Santa Cruz Biotechnology, Santa Cruz, CA, USA. When they were at 80% to 90% confluence, Hep-2 cells were transfected with the plasmids using the Lipofectamine 2000 according to the manufacturer's protocol. The stable clones (Hep-2/sCLU-shRNA and control Hep-2/shRNA) were selected by culturing transfected cells in the presence of 400 μgml^-1^ G418 (InvivoGen) for 10 days. Stable pooled populations of Hep-2/sCLU-shRNA and control Hep-2/shRNA cells were maintained in culture using 200 μgml^-1^ of G418. The knockdown effect was verified by RT-PCR and Western blot analysis.

### Western blots

The cells were washed after transfection with sCLU-siRNA or control siRNA for 48 h in the Hep-2 cells. Cell lysates were prepared by applying 400 μL lysis buffer (10 mmol/L Tris–HCl (pH 8.0), containing 150 mmol/L NaCl, 1 mmol/L phenylmethylsulfonyl fluoride, and 1% Triton X-100) to confluent cells grown in 60 mm dishes. A total of 25 μg protein was loaded on 4% to 20% Novex Tris-Glycine gradient denaturing polyacrylamide gels (Invitrogen) in a 1% SDS-PAGE buffer (1 g/L SDS, 3 g/L Tris base and 14.4 g/L glycine). Proteins were transferred to polyvinylpyrrolidine difluoride membranes electrophoretically and incubated overnight at 4°C in Blotto (5% dry milk in 1% TBS (0.9% NaCl, 10 mmol/L Tris (pH 7.4), and 0.5% MgCl_2_)). Membranes were incubated for 60 minutes at room temperature with anti-sCLU antibody (Santa Cruz Biotechnology) overnight at 4°C. The membrane was incubated with a 1:4,000 dilution of horseradish peroxidase-linked anti-mouse secondary antibodies. The immune complexes were detected using ECL Western blot detection reagents. The membranes were stripped of bound antibody and reprobed with an anti-β-actin antibody to confirm equal loading of the samples.

### RNA extraction and RT-PCR

After transfection with sCLU-siRNA or control siRNA for 48 h in the Hep-2 cells, the total RNA was extracted from the cells using Trizol reagent (Invitrogen). cDNAs were synthesized using a ThermoScript RT-PCR system according to the manufacturer's instructions (Invitrogen). Two oligonucleotides (5′-AGATCAGCGCCTGAGAAGCT-3′ and 5′-GGGACCAGTGTACCTTCTCG-3′) were used as specific primers to amplify the human sCLU sequence. The human β-actin cDNA fragments were amplified by the primers 5′-GCTCGTCGTCGACAACGGCT-3′ and 5′-CAAACATGATCTGGGTCATCTTCTC-3′. The PCR products were separated on 2% agarose gels and the density of each product was measured.

### MTT assays

MTT was dissolved in phosphate-buffered saline (PBS) and adjusted to a final concentration of 5 mg/ml. For MTT assays, Hep-2 cells transfected with sCLU-siRNA or control siRNA for 48 h (4 × 10^3^/well). Then 20 μl MTT was added to each well. After an additional 4 h at 37°C, culture media was removed and 150 μl DMSO was added. Plates were swirled gently in the dark for 10 minutes at room temperature (RT). Absorbance values at 490 nm (A490) for each well in 24 to 96 h were then measured using an enzyme-linked immunosorbent detector. Based on these data, cell growth inhibition ratios were calculated according to the following formula:

$$\begin{array}{l} \mathrm{cell}\;\mathrm{growth}\;\mathrm{inhibition}\;\mathrm{ratio}\left(\%\right)\\ \;=\left(\mathrm{control}\;\mathrm{group}\;A\;\mathrm{value}–\mathrm{experimental}\;\mathrm{group}\;A\;\mathrm{value}\right)/\\ \;\mathrm{control}\;A\;\mathrm{value}\times \% \end{array}$$

### Annexin V-FITC/PI double-staining

A total of 3 × 10^5^ Hep-2 cells, which was transfected with sCLU-siRNA or control siRNA for 48 hs, was collected and washed with PBS. Five microliters of Annexin V-FITC and 4 μl of PI were added and incubated at room temperature for 15 minutes and cells were then analyzed by flow cytometry. Cells were collected by the CellQuest system (USA) and the apoptosis rate was analyzed with the ModFit LT for Mac V3.0 software system (Verity Software House, USA). The apoptosis detection kit was purchased from Beijing Baosai Technology Company (China).

### Matrigel invasion assays

*In vitro* invasion assays were performed by using a 24-well invasion chamber coated with Matrigel (Becton Dickinson). Hep-2 cells were transfected with sCLU-siRNA or control siRNA for 24 h, then were trypsinised, washed with PBS, suspended in DMEM containing 5% bovine serum albumin (BSA), and plated in the invasion chamber (3 × 10^4^ cells per well). The lower chambers were filled with DMEM containing 5% BSA with 2.5% fetal bovine serum. After 24 h, the cells remaining in the upper chamber were removed by scraping, whereas the cells that invaded through Matrigel were fixed and stained by using 0.5% crystal violet in methanol. All invading cells were counted by microscopic visualisation. The cell invasions were performed by extracting the crystal violet dye with dimethylsulphoxide followed by spectrophotometry at 590 nm. All analyses were performed in triplicate.

### Wound healing assay

Hep-2 cells transfected with sCLU-siRNA or control siRNA for 48 h were grown to confluence in 35-mm tissue culture dishes. Cell monolayers were scratched using a micropipette tip, and floating cells were removed by extensive washing with DMEM. Photographs of the wounded area were taken immediately after making the scratch (0 h time point) and after 20 h to measure the migration rate of cells into the wounded area. At least 15 different fields were randomly chosen across the wound length.

### Subcutaneous xenografted tumor model

All the experiments were performed under the approval of the Animal Experimentation Committee of Linyi city’s hospital. Female SCID mice, each four to six weeks old, were obtained from Shandong University. All animals were maintained in a sterile environment and cared for within the laboratory animal regulations of the Ministry of Science and Technology of the People’s Republic of China. Full details of the study were approved by the ethics committee at the Junan County Hospital. A suspension of 2 × 10^6^ cells (Hep-2 cells, sCLU-shRNA/Hep-2 cells and control shRNA/Hep-2 cells, respectively) in 50 μL volume was injected subcutaneously into the left posterior flank of mice by use of a 1-cc syringe with a 27½-gauge needle. Tumors were grown for 28 days. Tumor size was measured using Vernier calipers and tumor volume was calculated as 0.5 × the longest diameter × width^2^. The percentage of tumor inhibition was calculated according to the formula [1 - (T/C)] × 100, where T and C represent the mean tumor volumes of the treatment group and the control group, respectively. Immunohistochemical staining and terminal deoxytransferase-mediated dUTP nick end labeling (TUNEL) staining for tumor tissues were done according to the method presented below.

### Immunohistochemistry *in vivo*

The primary tumor was harvested, fixed in 4% formalin and embedded in paraffin. Three-micrometer-thin tissue sections were obtained and stained for sCLU according to the manufacturer’s instructions.

### *In situ* TUNEL assay

TUNEL staining for tumor tissue was based on the protocol of the Dead End Colorimetric TUNEL System (Promega). Briefly, cryostat sections were fixed in 4% paraformaldehyde, and *in situ* TUNEL assay was done as described by the manufacturer (Roche Molecular Biochemicals, Manheim, Germany). The number of apoptotic cells was determined in relation to the total number of cells.

### Lung metastasis model of nude mouse

In order to induce lung metastasis, Hep-2 cells, sCLU-shRNA/Hep-2 cells and control shRNA/Hep-2 cells (1 × 10^6^ cells/mouse) in 100 μL of PBS were injected into the mice’s tails for 21 consecutive days. After 21 days, the mice were sacrificed, and the lungs were fixed in Bouin’s solution. Lung tumors were then analyzed by obtaining a surface naked tumor count.

### Statistical assay

All data were displayed by mean ± standard deviation (SD) and produced by SPSS11.5 statistical software (SPSS Inc., Chicago IL, USA). Variance analysis was performed for the comparison of multiple groups. Group-group comparison was carried out with the Student-Newman-Keuls method (SNK), and a *P* <0.05 was considered a statistically significant difference.

## Results

### Knockdown of sCLU results in specific attenuation of sCLU

**Figure 1:**
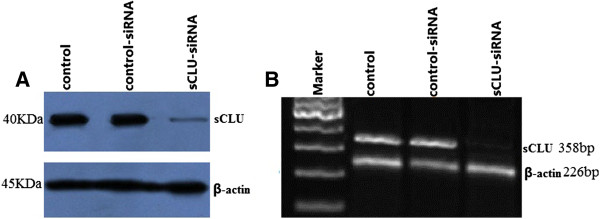
**Effect of sCLU-siRNA transfection on mRNA and protein expressions of sCLU. A**. Western immunoblot analysis of sCLU expression in Hep-2 cells transfected with sCLU-siRNA. Blots were re-probed with β-actin antibody to analyze the equal loading of proteins. **B**. Semiquantitative RT-PCR analysis shows mRNA expression of sCLU in Hep-2 cells transfected with sCLU-siRNA. Scrambled siRNA was used as a control in parallel. β-actin was used as an internal control. This is a representative example with mean densitometric values from triplicate blots. ^*^, *P* <0.05 versus control siRNA transfectants. sCLU, Clusterin; siRNA, small interference RNA.

sCLU-siRNA and control siRNA were transiently transfected into Hep-2 cells by use of Lipofectin 2000 for 48 h. The cells were collected and protein extracts were made from the cytoplasm. Protein levels of sCLU were analyzed by Western blot. As shown in Figure 1A, sCLU protein was overexpressed in the Hep-2 cells, in the sCLU-siRNA transfected Hep-2 cells, and sCLU protein was completely inhibited; however, control siRNA transfection did not induce a significant change in expression of sCLU in the Hep-2 cells. A sCLU mRNA assay by RT-PCR has the same results as shown above (Figure 1B).

### Knockdown of sCLU inhibited proliferation and stimulated apoptosis of Hep-A2 cells

**Figure 2:**
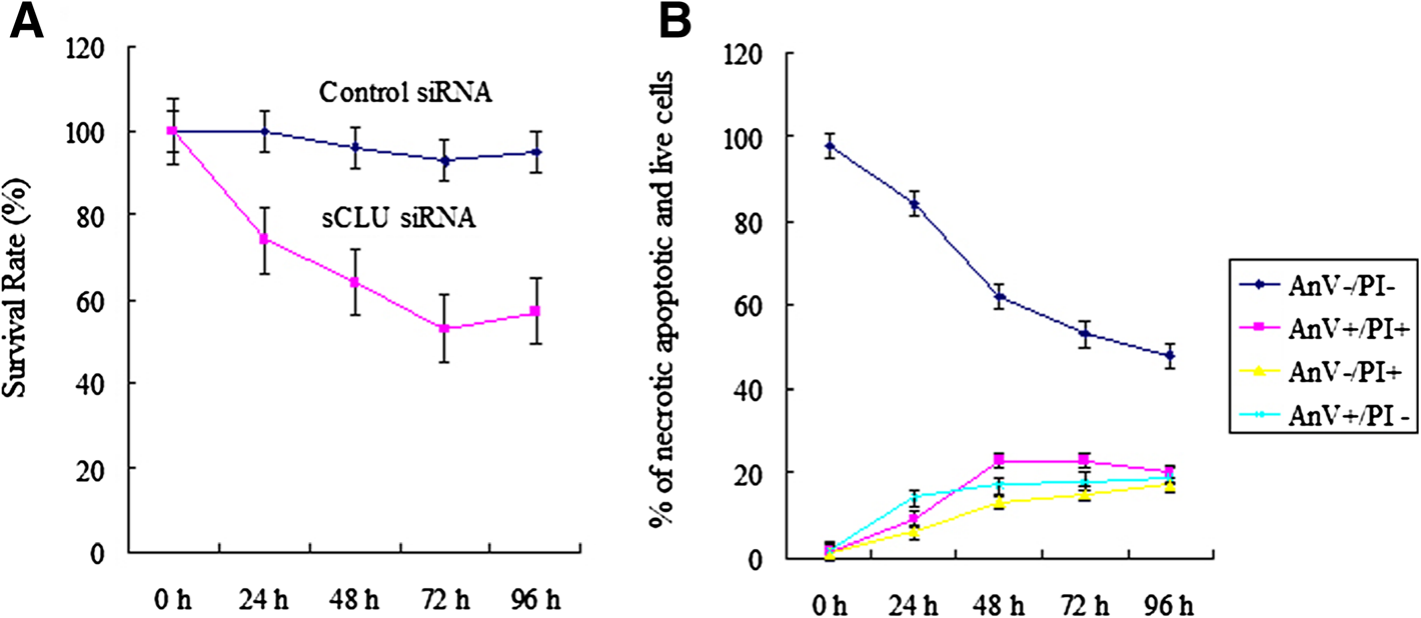
**sCLU siRNA-mediated suppression of proliferation and increase of apoptosis in the Hep-2 cells. A**: The effect of sCLU silencing on proliferation in Hep-2 cells by MTT assay. The values represent the mean ± SE, n = 3. **B**: cells were stained with AnV and PI, analyzed by flow cytometry, and the percentages of cells in groups AnV^+^/PI ^-^ (early apoptosis), AnV^-^/PI^+^ (early necrosis), AnV^+^/PI^+^ (mixture of apoptosis associated with secondary necrosis and late necrosis), and AnV^-^/PI^-^ (live cells) were measured. The values represent the mean ± SE, n = 3.

As shown in Figure 2A, knockdown of sCLU by sCLU-siRNA significantly inhibited Hep-2 cell growth determined by MTT assay over four days. These results indicate that sCLU silencing is overall an effective inhibitor of Hep-2 cell growth. Knockdown of sCLU-induced cell death was further confirmed by flow cytometry (Figure 2B). AnV^+^/PI^-^ represents cells with early apoptosis - AnV^-^/PI^+^ with early necrosis, AnV^+^/PI^+^ with a mixture of apoptosis associated with secondary necrosis and late necrosis, and AnV^-^/PI^-^ with live cells. No significant difference was found between Hep-2 cell and control siRNA transfected Hep-2 cells (data not shown).

### Knockdown of sCLU inhibits invasion of Hep-2 cells *in vitro*

**Figure 3:**
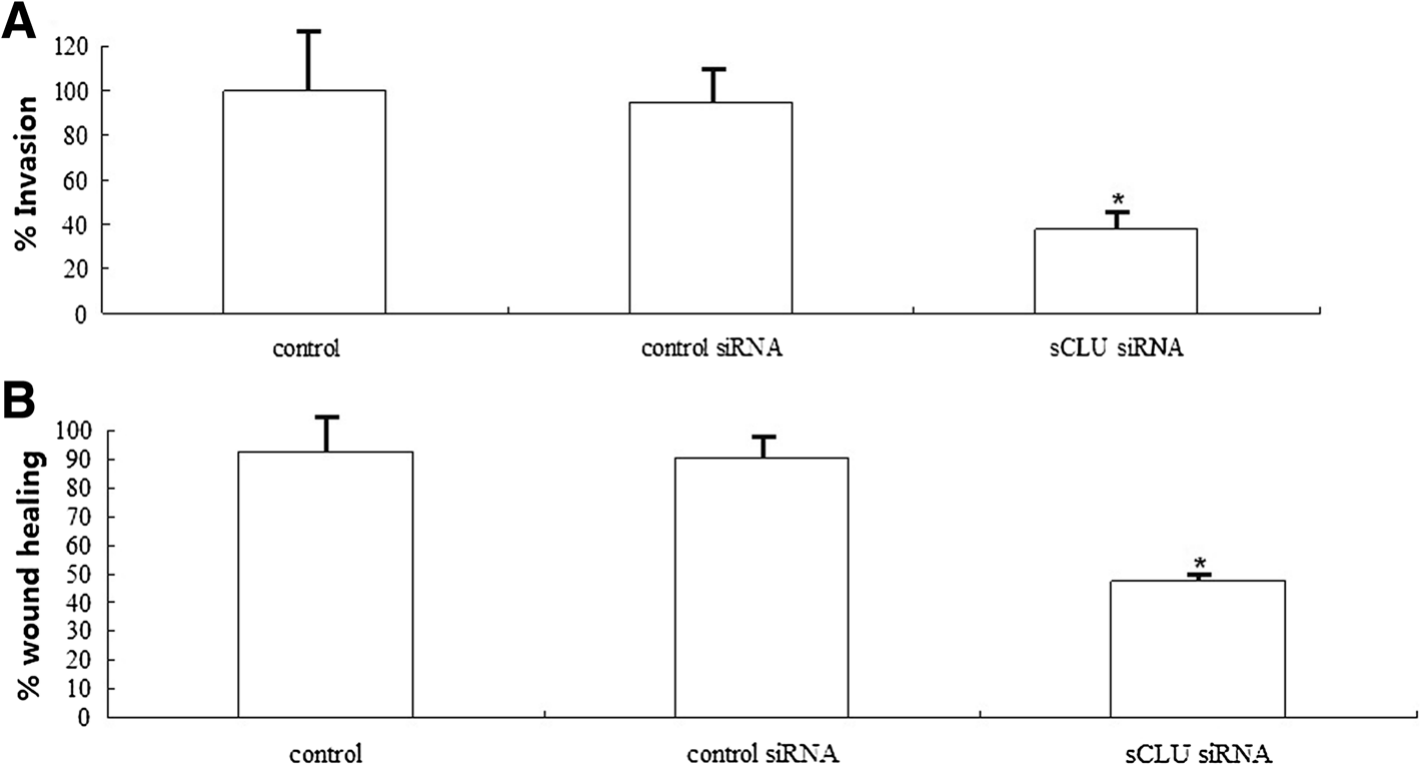
**sCLU silencing inhibits invasion of human Hep-2 cells in vitro. A**. The bar shows the number of migratory cells transfected with sCLU siRNA and control siRNA. At bottom is the quantification of migratory cells in control siRNA and sCLU siRNA transfected Hep-2 cells. The experiment was done in triplicate and the value obtained from control siRNA transfected cells was set as 100% migration. **B**. A wound was scratched in a confluent cell layer of Hep-2 cells. Cells were either transfected with control siRNA (control) or sCLU siRNA for 24 h. The percentage of coverage after 24 h of wound healing is depicted in the graphs for Hep-2 cells. Knockdown of sCLU significantly reduces wound healing in Hep-2 cells. Significance was determined with a Student's *t*-test vs control, ^*^P <0.05. sCLU, Clusterin; siRNA, small interference RNA.

We tested whether sCLU knockdown affected the invasion capabilities of Hep-2 cells by using an *in vitro* invasion assay. Cells were seeded in the upper part of a Matrigel-coated invasion chamber in a 5% FCS concentration. After 24 h, the cells remaining in the upper chamber were removed by scraping, whereas the cells that invaded through Matrigel were fixed and stained by using 0.5% crystal violet in methanol. In sCLU-silenced Hep-2 cells, invasion was significantly reduced (Figure 3A).

To analyze the effect of sCLU knockdown on wound healing, a scratch assay was performed with Hep-2 cells. In control siRNA transfected Hep-2 cells, 90.6% (±11.74) of the wound was closed after 24 hours. In contrast, in sCLU siRNA transfected Hep-2 cells, only 48.4% (±10.2) of the wound was closed (Figure 3B).

### Effects of sCLU shRNA on xenograft tumor growth

**Figure 4:**
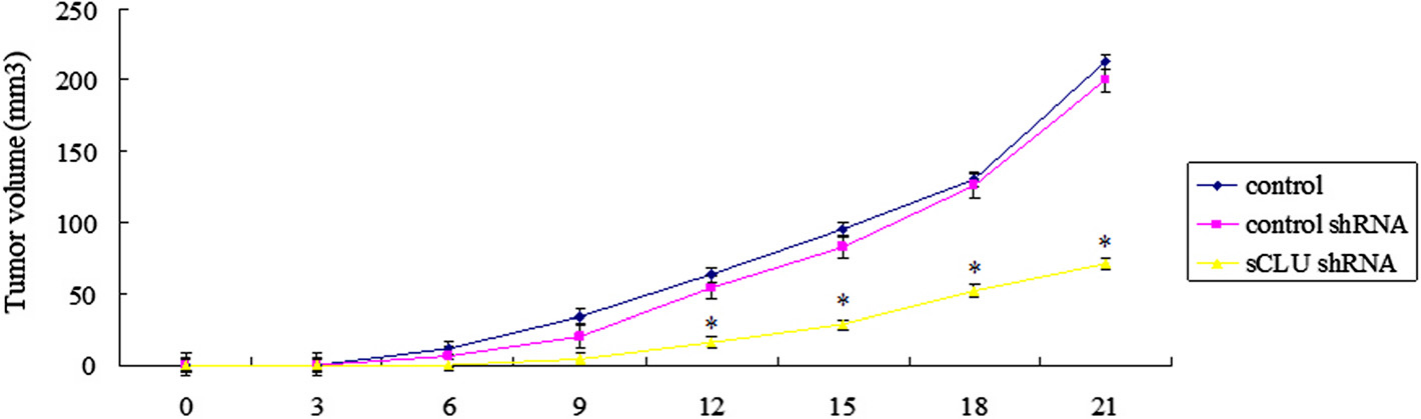
**The growth curve of subcutaneous tumors.** Tumor growth was strongly suppressed following sCLU shRNA transfection compared with control shRNA and control. Asterisks indicate *P* < 0.01. sCLU, Clusterin; shRNA, short hairpin RNA.

As shown in Figure 4, on the 21^st^ day, the average tumor volume in each group was as follows: controls, 213.5 ± 10.7 mm^3^; control shRNA, 200.0 ± 9.6 mm^3^; and sCLU shRNA, 71.3 ± 7.8 mm^3^. The tumor size in group sCLU shRNA mice was significantly smaller compared to the control, with a tumor inhibition ratio of 72.3%. Statistical analysis revealed no significant difference between the negative control shRNA and the control group (*P* > 0.05), thereby suggesting that the control plasmids had no obvious side effect on tumor growth.

### Apoptosis in xenograft tumors

**Figure 5:**
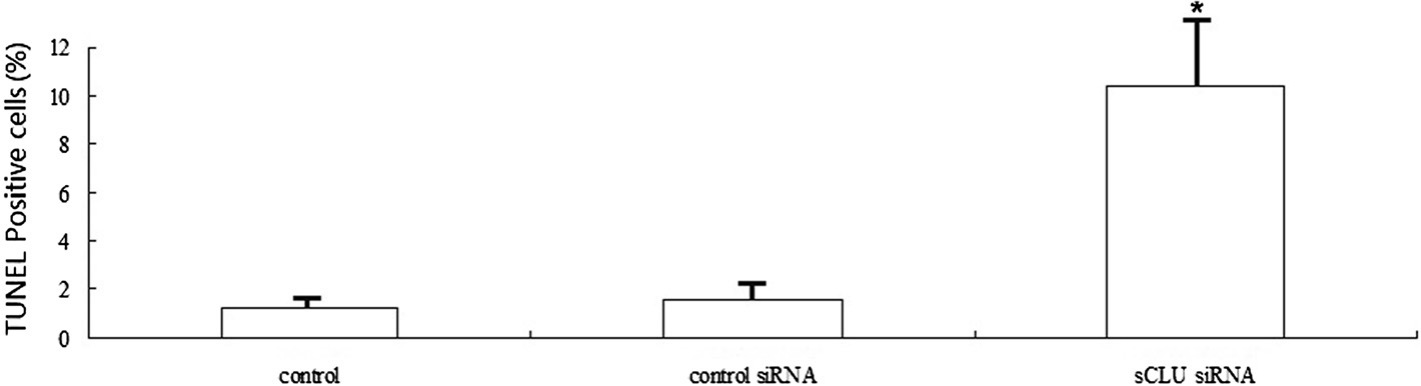
**Apoptosis in subcutaneous tumors.** In the tumors treated with sCLU shRNA, significant TUNEL-positive cells were found. However, only a few apoptotic cells were found in the tumors treated with the control shRNA and the saline. The apoptotic index of sCLU shRNA was much higher than that of the control groups. Asterisks indicate *P* <0.01. sCLU, Clusterin; shRNA, short hairpin RNA; TUNEL, terminal deoxytransferase-mediated dUTP nick end labeling.

In the tumors transfected with sCLU-shRNA, 12% of tumor cells were TUNEL-positive (Figure 5). However, only 1 to 2% of apoptotic cells were found in the tumors transfected with the control shRNA and the saline control. There were significantly more apoptotic cells in the sCLU-shRNA group than in the control shRNA and saline-treated groups.

### The expression of sCLU in xenograft tumors

In the tumors transfected with the control shRNA, the sCLU protein was significantly higher (data not shown). However, the sCLU protein was significantly decreased in the majority of the tumor cells from mice that were transfected with sCLU shRNA by immunohistochemical assay (data not shown).

### sCLU silencing inhibits Hep-2 cells lung metastasis

**Figure 6:**
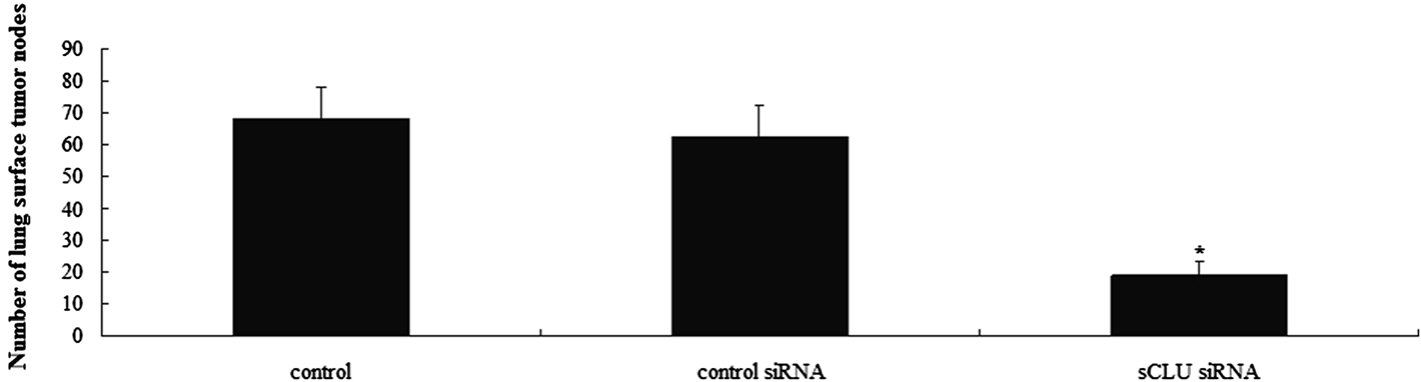
**The effect of sCLU shRNA on lung metastasis.** This is a representative histogram showing the surface tumor number in three groups. Each bar represents mean ± SE; *, *P* <0.01. All experiments were repeated three times with similar results. sCLU, Clusterin; shRNA, short hairpin RNA.

In the sCLU shRNA transfected groups, lack of sCLU had a significant effect on development of lung metastasis with a 66.4% reduction in surface tumor number (control shRNA groups’ median tumor number = 62.4 ± 6.7; sCLU shRNA transfected group’s median tumor number =18.6 ± 4.3; Figure 6).

## Discussion and conclusions

RNA interference (RNAi) is a sequence-specific post-transcriptional gene silencing tool [22]. The process of gene-specific silencing through destruction of its mRNA transcript can be triggered by endogenous or exogenous small interfering RNAs (siRNAs) [23]. Long double-stranded mRNAs derived from endogenous gene transcription or transfected transgene plasmids present in the cytoplasm can trigger the cleavage activity of the intracellular enzyme, Dicer, to cut mRNA into 19-nucleotide pairs with two nucleotide overhangs at both 3’-ends, called small interfering RNA (siRNA) [24]. Gene silencing using siRNA has several advantages intrinsic to RNAi, such as its high specificity, intrinsic biological response [25], and more efficient and specific silencing effects with lower dosing requirements, compared to antisense-based gene silencing [26]. However, single-dose siRNA silencing effects are transient (up to five days in dividing cells) [23] and lipid-based siRNA delivery complexes can be rapidly removed from circulation by the liver, and lack tissue/cell specificity. Therefore, we used siRNA against sCLU to study the effect of sCLU *in vitro* for its short duration.

Apoptosis plays an essential role as a protective mechanism against carcinogenesis by eliminating genetically damaged cells - initiated cells as well as those cells that have progressed to malignancy. Our presented results clearly demonstrate that sCLU mRNA and protein is amenable to specific siRNA-induced degradation *in vitro*. Moreover, it is evident that the primary function of sCLU in Hep-2 cells is anti-apoptotic. In Hep-2 cells, sCLU knockdown resulted in significant growth retardation, higher rates of endogenous apoptosis and reduced metastatic potential. Interestingly, it was recently shown that sCLU overexpression into human renal cell carcinoma cells enhances their metastatic potential [12]. Whether sCLU overexpression could enhance the metastatic potential and growth potential in Hep-2 cells needs further investigation.

Viral vector-based delivery is consistently associated with vector-based shRNA production systems, a DNA-based strategy to encode and obtain host-synthesized shRNAs *in situ*. These shRNAs can be further intracellularly processed into siRNA by Dicer. The viral vector-based shRNA strategy has the potential of being able to provide stable, enduring gene silencing. Gene therapy can in principle continuously generate siRNA [24]. Therefore, we used shRNA against sCLU to study the effect of sCLU *in vivo* for its long duration. Our presented results clearly demonstrate that shRNA targeted to sCLU mRNA can inhibit the growth of laryngeal cancer Hep-2 cells by 73% *in vivo*, and significantly more apoptotic cells in the sCLU-shRNA targeted groups, proved sCLU’s anti-apoptotic potential. Furthermore, shRNA targeted to sCLU exhibited a significant lung metastasis model. In the clinical course of laryngeal carcinomas, lung metastasis is a very unfavorable development that frequently occurs in patients with laryngeal carcinomas. However, the mechanism of how sCLU works is still uncertain.

In summary, sCLU is a target for therapeutic inhibition in laryngeal carcinomas. The siRNAs (shRNAs) used in this study are potent tools for modulating the sCLU gene expression and they may ultimately develop into attractive antitumor therapeutics.

## Abbreviations

BSA: bovine serum albumin; DMEM: Dulbecco’s modified Eagle’s medium; FITC: fluorescein isothiocyanate; HEp2: human epithelial type 2 cells; LSCC: Laryngeal squamous cell carcinomas; PBS: phosphate-buffered saline; PI: propidium iodide; RNAi: RNA interference; RT: room temperature; sCLU: Clusterin, a secreted heterodimeric disulfide-linked glycoprotein; shRNA: short hairpin RNA; siRNA: small interference RNA; TUNEL: Terminal deoxytransferase-mediated dUTP nick end labeling stains.

## Competing interests

The authors declare that they have no competing interests.

## Authors’ contributions

QW and LZ performed the research and contributed to data analysis and manuscript writing. ZL, WC and QS performed the research and contributed with analytical tools. LZ designed the research and contributed to data analysis. QW designed the research and contributed to data analysis and manuscript writing. All authors read and approved the final manuscript.
